# Targeting histone deacetylase and NFκB signaling as a novel therapy for Mucoepidermoid Carcinomas

**DOI:** 10.1038/s41598-018-20345-w

**Published:** 2018-02-01

**Authors:** Vivian P. Wagner, Manoela D. Martins, Marco A. T. Martins, Luciana O. Almeida, Kristy A. Warner, Jacques E. Nör, Cristiane H. Squarize, Rogerio M. Castilho

**Affiliations:** 1 Laboratory of Epithelial Biology, Department of Periodontics and Oral Medicine, University of Michigan School of Dentistry, Ann Arbor, MI 48109-1078 USA; 2 Experimental Pathology Unit, Clinics Hospital of Porto Alegre, Federal University of Rio Grande do Sul, Porto Alegre, RS 90035-003 Brazil; 3 Department of Oral Pathology, School of Dentistry, Federal University of Rio Grande do Sul, Porto Alegre, RS 90035-003 Brazil; 4 Department of Restorative Sciences, University of Michigan School of Dentistry, Ann Arbor, MI 48109 USA; 5 Comprehensive Cancer Center, University of Michigan, Ann Arbor, MI 48109 USA; 6 Department of Otolaryngology, Medical School, University of Michigan, Ann Arbor, MI USA; 7 Department of Biomedical Engineering, University of Michigan College of Engineering, Ann Arbor, Michigan USA

## Abstract

Malignancies from the salivary glands are rare and represent 11% of all cancers from the oropharyngeal anatomical area. Mucoepidermoid Carcinomas (MEC) is the most common malignancy from the salivary glands. Low survival rates of high-grade Mucoepidermoid Carcinomas (MEC) are particularly associated with the presence of positive lymph nodes, extracapsular lymph node spread, and perineural invasion. Most recently, the presence of cancer stem cells (CSC), and the activation of the NFκB signaling pathway have been suggested as cues for an acquired resistance phenotype. We have previously shown that NFκB signaling is very active in MEC tumors. Herein, we explore the efficacy of NFκB inhibition in combination with class I and II HDAC inhibitor to deplete the population of CSC and to destroy MEC tumor cells. Our finding suggests that disruption of NFκB signaling along with the administration of HDAC inhibitors constitute an effective strategy to manage MEC tumors.

## Introduction

Salivary gland cancer (SGC) annual incidence rates vary between 0.05 to 2 new cases per 100,000 habitants^1^. The relative small incidence of SGC allied with a significant clinical and biological heterogeneity poses critical challenges on the study of these diseases. SGC can present slow progression^1^ leading to low survival rates on long-term analysis^2^. Mucoepidermoid carcinoma (MEC) represents the most common SGC^3–5^. This tumor is graded histologically according to its architectural and morphological features^6^. The prognosis for low-grade MEC is considerably good, with 5-year survival rates reaching rates above 90%. The real problem relies on high-grade tumors and advanced cases. In these conditions, the 5-year survival rates considerably drop to 51% for high-grade tumors and, more alarmingly, to 32% upon nodal involvement and 26% for distant metastasis^7^. Currently, all systemic approaches to MEC are considered merely palliative being cisplatin and cisplatin-based regimens the most frequently used. Other systemic drugs such as Methotrexate, Paclitaxel, Doxorubicin have also been evaluated in clinical trials (reviewed in^8^). Response rates vary between 10% and 70%, however, in most trials, only a few patients are enrolled and the follow-up time is short. Therefore, there is no evidence that systemic therapies can significantly improve survival of MEC patients.

MEC treatments are often a transposition of protocols tailored to squamous cell carcinomas^9^. The lack of pre-clinical models resulted in a poor biological understanding of the disease. Recently, MEC cell lines were established at the University of Michigan School of Dentistry^10,11^ enabling better characterization of the biological response of MEC cells to new therapeutic approaches. An important issue associated with tumor resistance in several types of solid tumors is the presence of cancer stem cells (CSC). This subpopulation of highly tumorigenic neoplastic cells was recently described in MEC^11^. CSC are recognized by their potential to initiate and maintain tumor growth and progression in several cancers. It has been demonstrated that CSC can endure G0 cell cycle, which provides them a quiescent profile that allows these cells to evade conventional treatments that target proliferative cells^12^. Other factors contribute to their resistant profile, such as the perivascular niche^13^, capacity to modulate DNA repair systems^14^ and chromatin status^15^. Our group observed alarming results regarding the effect of conventional therapies in MEC CSC population. We detected that cisplatin induces the accumulation of CSC^16^, while ionizing radiation doesn’t significantly impact the CSC population of a metastatic MEC cell line^17^.

In order to find new and highly efficient treatments for MEC, it’s of paramount importance to identify molecular signatures and signaling pathways associated with tumor resistance. We recently demonstrated that intrinsic NFκB activation triggers MEC resistance to ionizing radiation^17^. Moreover, our group has previously shown that NFκB activation also mediates cisplatin resistance through histone modifications^18^. We had promising results regarding sensitization of MEC tumor cells by targeting NFκB or histone acetylation followed by conventional therapies. We demonstrated that Emetine, an NFκB inhibitor, and Vorinostat (Suberoylanilide Hydroxamic Acid_SAHA), an HDAC inhibitor, are capable of increasing the efficiency of ionizing radiation^17^ and cisplatin^16^, respectively. Emerging evidence underscores the importance of targeting multiple pathways involved in tumor progression and resistance to therapy to completely eradication of cancer. These studies suggest that a small subpopulation of tumor cells exhibiting resistance to one specific pharmacological agent is always present in advanced tumors^19–21^. Bozic *et al*.^21^, used a mathematical approach to predict tumor control in an example of a skin melanoma showing eight metastatic lesions. According to these authors, there is a 0% chance of disease control using a single drug. Remarkably, the likelihood of treatment success can rise to 88% when two drugs with different targets are combined^21^.

Taking into consideration the benefits of combined therapy, we decided to explore the inhibitory benefits of targeting HDAC and NFκB combined compared with the single administration of Vorinostat and Emetine. We observed that Emetine alone is effective in reducing tumor cells, whereas Vorinostat efficiently disrupted the population of CSC, but failed in significantly reducing the total number of tumor cells. When combined, however, Emetine plus Vorinostat effectively reduced CSC and colony forming tumor cells.

## Results

### Single dose of Emetine disrupts MEC colony formation

Malignant cells usually present elevated NFκB activity^22^ leading to increased cell survival through deactivation of apoptotic pathways^23^. The NFκB pathway is activated by a pro-inflammatory stimulus, which triggers IKK complex activation, followed by IκB-α phosphorylation. These events allow NFκB to translocate to the nucleus where it becomes active acting as a transcription factor^24,25^. Recently, we demonstrated that MEC tumor samples present high levels of nuclear NFκB compared to normal salivary gland tissues^17^.

Platinum-based regimens are most frequently employed to treat advanced cases of SGC^9^, however patient survival remains poor^26^. Emetine is a FDA-approved drug that has been used for many decades to treat protozoan infections and amoebiasis^27^. Recently, it has been shown that Emetine has high inhibitory effects over the NFκB pathway through the phosphorylation of IκB-α^28^. We have further dissected the pathway and found that Emetine is an efficient inhibitor of the IKK-β that result in the phosphorylation of IκB-α and NFκB degradation^17^.

**Figure 1:**
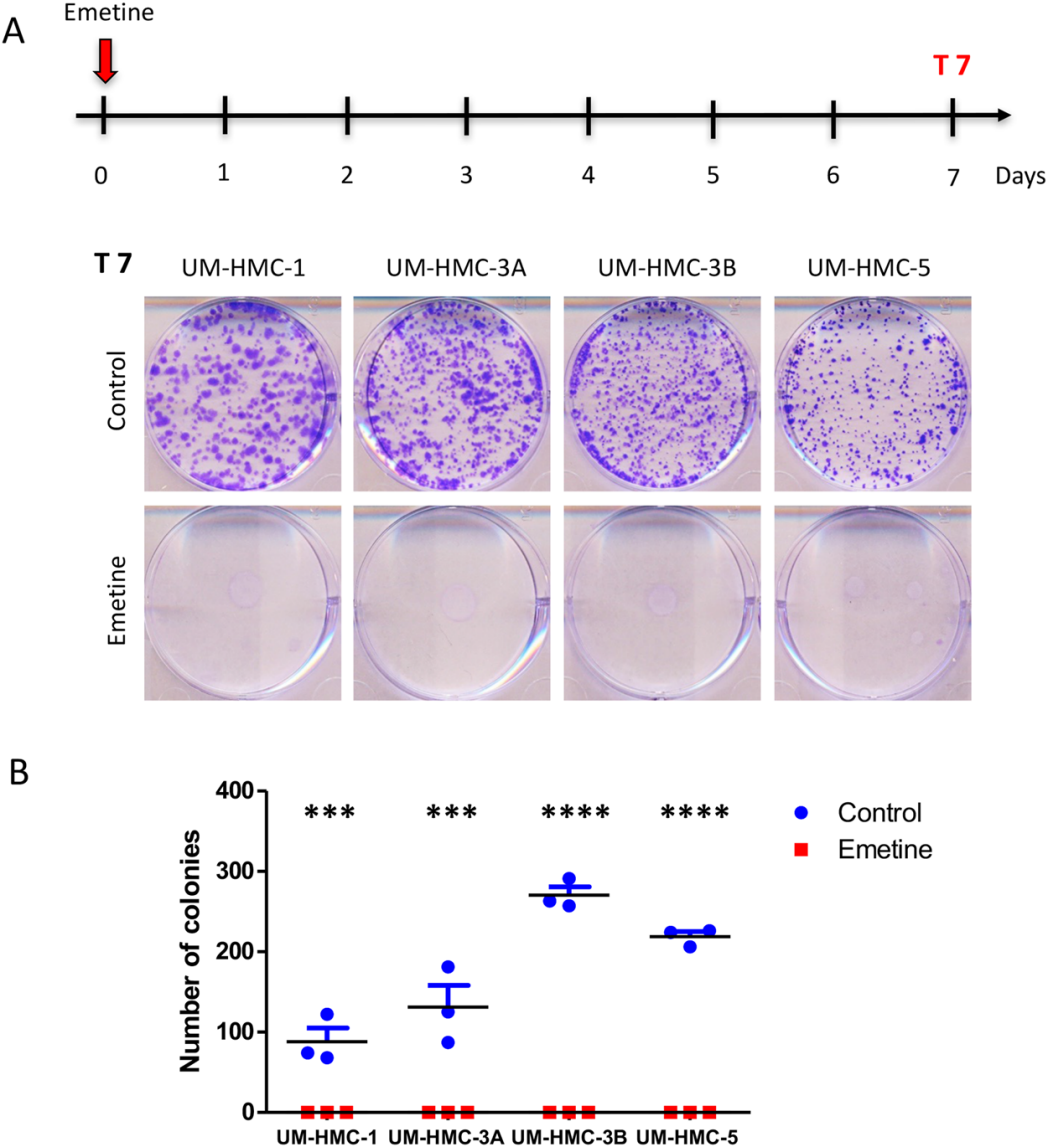
A single dose of Emetine is capable of abrogating reproductive viability of MEC cells. (**A**) Schedule of Emetine administration followed by clonogenic assay. A single dose of Emetine (determined by each cell line IC50) was administrated on day 0. Colony formation was assessed on day 7. Representative images of colonies fixed and stained with 0.1% crystal purple. Note that Emetine treatment led to a complete disruption of colony formation in all four lineages. (**B**) Colony quantification (colonies having >50 cells were counted as surviving colonies) revealed that UM-HMC-3B and UM-HMC-5 presented a higher number of colonies. After Emetine administration, the number of colonies was reduced to zero in all cell lines.

Recent studies verified that Emetine is capable of stimulating apoptosis of pancreatic^29^, leukemic^30^, and ovarian carcinoma cells^31^ and inducing cell growth arrest in bladder cancer cells^32^. We have shown that administration of Emetine sensitizes MEC cells to ionizing. Further, Emetine has shown efficacy over the population of CSC^17^. However, the effects of Emetine as a single-agent drug in MEC had never been explored until now. Initially, we analyzed the ability of single dose of Emetine in disrupt tumor colony formation after 7 days of treatment (Fig. 1A). We observed that inhibition of the NFκB signaling at the specific Emetine IC50 of each tumor cell line (previously established by us^17^, led to a complete disruption of colony formation in all four tumor cell lineages (Fig. 1B) (***p < 0.001).

### Emetine reduces the number of MEC tumor spheres

**Figure 2:**
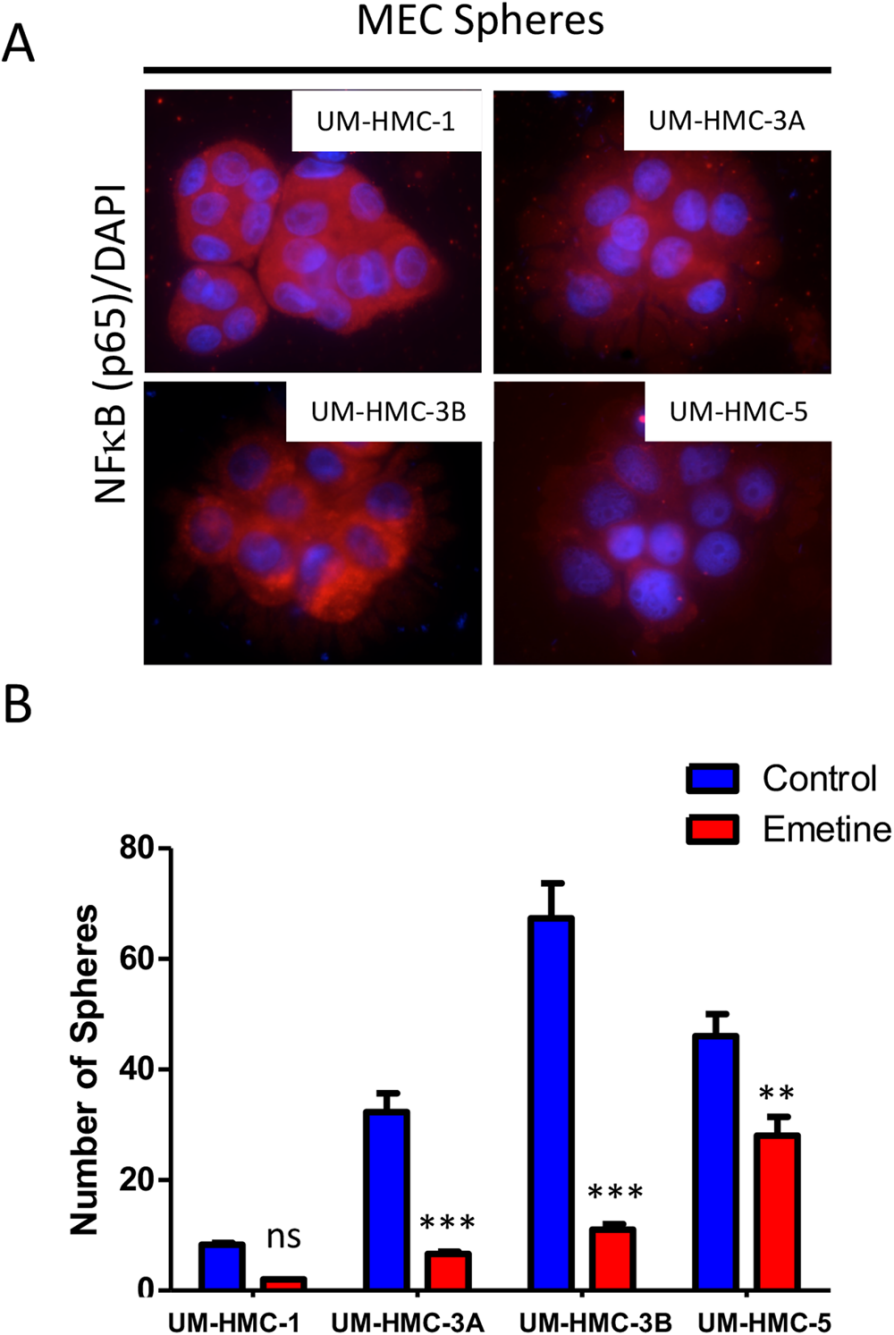
Spheres formation. (**A**) All MEC cell lines are capable of producing spheres under low attachment conditions. All spheres present NFκB nuclear expression (Immunofluorescence, stained with NFκB – TRITC and DNA content - Hoechst 33342, 100 × original magnification). (**B**) A single dose of Emetine reduced the number of spheres in all MEC cell lines. However, this decrease was not significant for UM-HMC-1 cell line.

Emerging evidence suggests that CSC are implicated in poor response to therapy leading to tumor recurrence and short overall survival^33,34^. Therefore, new therapeutic strategies need to take into consideration the effects of chemotherapy over the population of CSC. Our group has previously shown that MEC cell lines can generate tumor spheres upon culturing under ultra-low adhesion conditions^16^. In the present study, we observed that MEC spheres express significant levels of phosphorylated p65, suggesting a potential implication of NFκB signaling pathway activation on the formation of tumor spheres (Fig. 2A). Indeed, administration of Emetine resulted in a significant reduction in the number of MEC spheres suggesting the requirement of NFκB signaling to maintain tumor spheres integrity. Although all MEC cell lines derived spheres respond to Emetine, we achieved a significant reduction in the number of MEC spheres on 3 cell lines (UM-HMC-3A, 3B and 5). UM-HMC-1-derived MEC did not achieve statistical significance (Fig. 2B). NFκB inhibition has been associated with the downregulation of the stem cell markers Nanog and Sox 2^35^. Aligned with previous reports, we have recently shown that NFκB inhibition using Emetine resulted in tumor sensitization to radiation, in a process that involved the depletion of MEC CSC^17^.

### Vorinostat is highly efficient in depleting MEC tumor spheres

**Figure 3:**
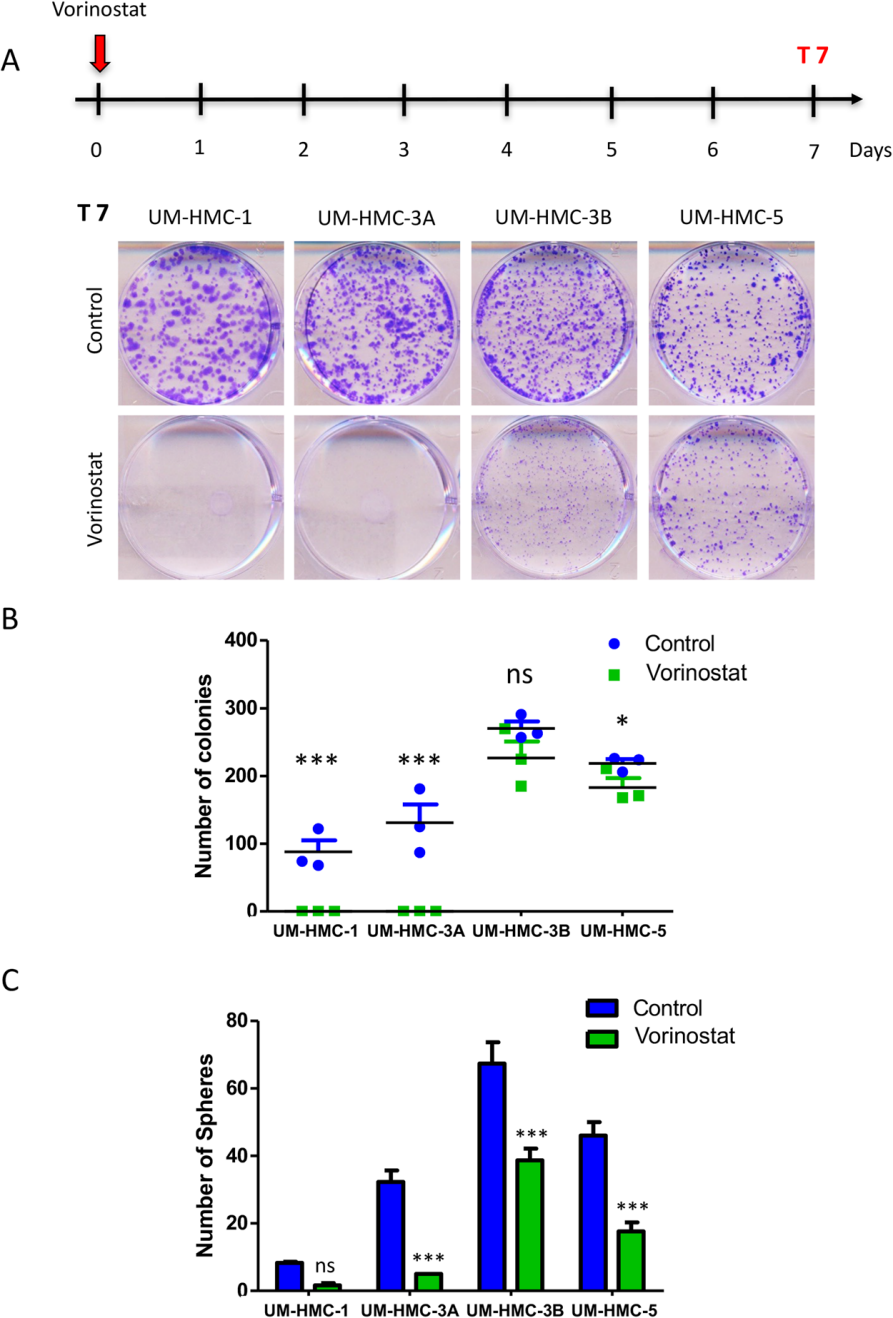
Effect of a single dose of Vorinostat on colony formation and spheres. (**A**) Schedule of Vorinostat administration followed by clonogenic assay. A single dose of Vorinostat (determined by each cell line IC50) was administrated on day 0. Colony formation was assessed on day 7. Representative images of colonies fixed and stained with 0.1% crystal purple. Note that Vorinostat treatment led to a complete disruption of colony formation only in UM-HMC-1 and UM-HMC-3A. (**B**) Colony quantification (colonies having >50 cells were counted as surviving colonies) revealed that after Vorinostat administration the number of colonies was reduced to zero only in HMC-1 and UM-HMC-3A, while the reduction of UM-HMC-3B and UM-HMC-5 was very discreet. (**C**) A single dose of Vorinostat reduced the number of spheres in all MEC cell lines. However, this decrease was not significant for UM-HMC-1 cell line.

Vorinostat is an FDA approved class I and II HDAC inhibitor in the treatment of T-cell lymphoma^36^. Vorinostat induces histone acetylation and impacts cancer cells by triggering growth arrest, differentiation, and apoptosis^37,38^. Our previous studies show promising results using Vorinostat to sensitize MEC and head and neck squamous cell carcinoma to cisplatin^16,18^. Herein, we decided to explore the effect of Vorinostat as a single-agent for MEC treatment. Initially, we evaluated the effects of histone acetylation on MEC colony formation. We observed that a single dose of Vorinostat was effective in completely depleting colony formation in UM-HMC-1 and UM-HMC-3A cell lines (***p < 0.001) (Fig. 3A and B). Interestingly, the metastatic cell line, UM-HMC-3B, Vorinostat had no impact on the number of colonies (ns - p > 0.05). Moreover, Vorinostat had a less significant impact in UM-HMC-5 regarding colony formation decrease (*p < 0.05) compared to Emetine (****p < 0.0001). We have recently demonstrated that p53 is highly active in UM-HMC-5 compared to others MEC cell lines^17^. The distinct profile of UM-HMC-5 might justify its increased resistance to Vorinostat once that p53 gain of function has been previously associated with mutations that can interfere with the process of apoptosis^39^. The discrepancy in the number of colonies after Vorinostat treatment suggest that MEC cell lines can respond differently to HDAC inhibition. Our group has demonstrated that MEC human samples present distinct stages of cellular differentiation and acetylation of histone H3 (lys9)^16^, which could justify the differences found regarding HDAC inhibition. Therefore, our results suggest that Emetine is more efficient in reducing the survival fraction of MEC cell lines when compared to Vorinostat.

Our group has shown promising results of Vorinostat interfering with the ability of head and neck malignancies to generate tumor spheres^15,16^. Therefore, we sought to investigate the effects of histone acetylation on the ability of MEC cell in generating tumor spheres. Interestingly, we found that single administration of Vorinostat was sufficient to significantly reduce the ability of MEC cells in generating tumor spheres. The only exception was UM-HMC-1 cell line that demonstrates the lowest potential in forming tumor spheres and the effect of Vorinostat, although evident, was not statistically significant (Fig. 3C).

### Vorinostat is more efficient than Emetine in reducing ALDH^+^ MEC cells

**Figure 4:**
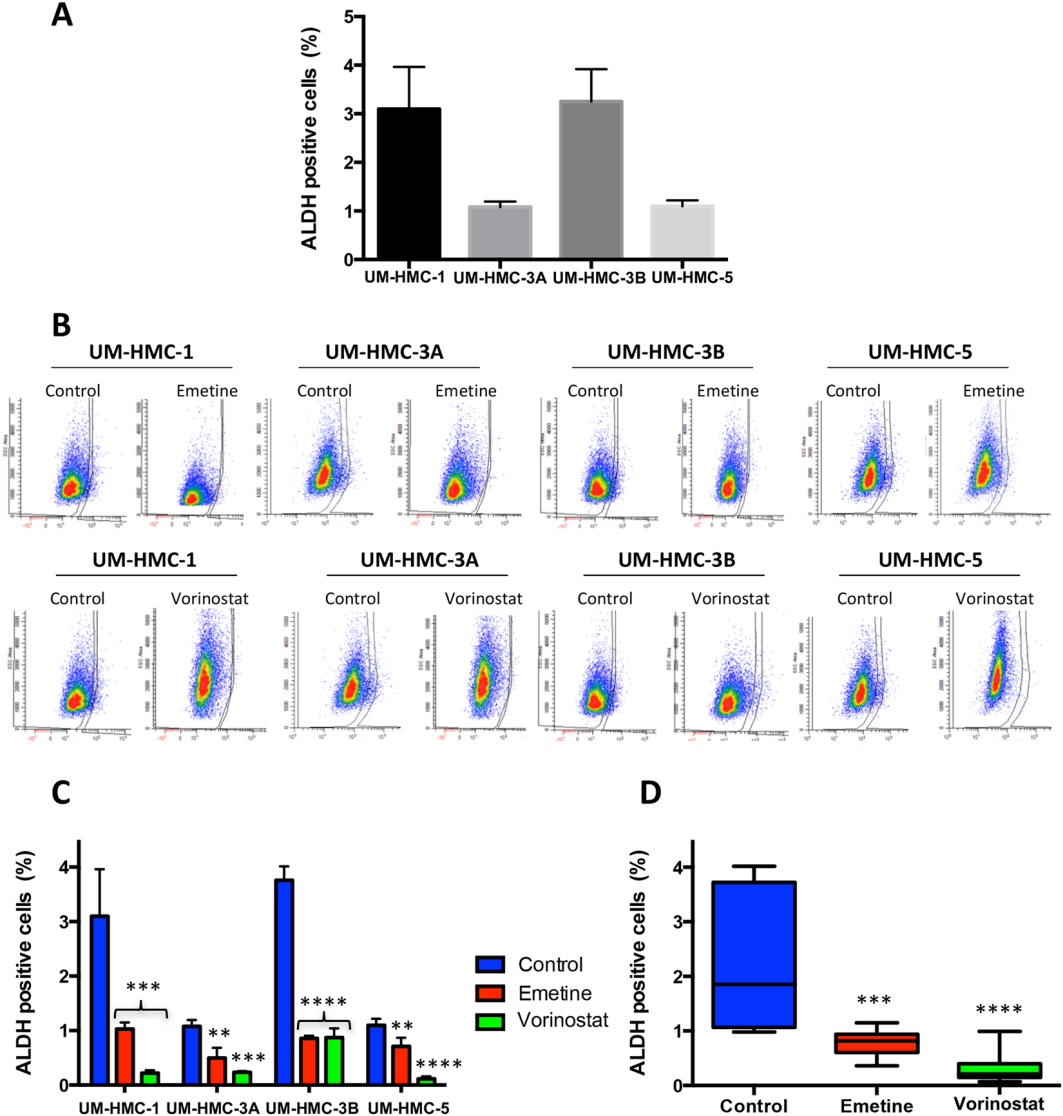
Effect of Emetine or Vorinostat as single agents in ALDH^+^ cell population. (**A**) MEC CSC population, assessed through ALDH enzymatic activity, ranges from 1.08 to 3.25%. Note that UM-HMC-1 and UM-HMC-2B present the higher percentages of CSC. (**B**) Effect of Emetine or Vorinostat on ALDH^+^ cells. (**C**) Emetine and Vorinostat significantly reduced ALDH^+^ cells in all cell lines. However, Vorinostat has a major impact in UM-HMC-1 and UM-HMC-3B. (**D**) Combining the results from all cell lines, we observed that Vorinostat was more efficient in reducing the CSC population of MEC cell lines.

Aldehyde dehydrogenase (ALDH) families of enzymes are cytosolic isoenzymes responsible for converting retinol to retinoic acid and also for oxidizing intracellular aldehydes, thereby conferring resistance to alkylating agents^40^. A previous study of Adams *et al*., demonstrated that enhanced tumorigenic potential of MEC cells, characteristic of CSC, can be assessed through ALDH activity^11^, corroborating with previous studies that show a similar profile in several malignant tumors such as head and neck squamous cell carcinoma^41^, breast cancer^42^ and colon cancer^40^. We have shown that head and neck cancers including squamous cell carcinomas, mucoepidermoid carcinomas, and adenoid cystic carcinomas do contain a small population of CSC expressing high levels of ALDH^11,15–17,43,44^. During our previous work, we have also show that the presence of CSC (ALDH bright) are directly associated with the resistance phenotype on head and neck cancers to chemo and radiotherapy. Therefore, accessing the CSC by the ALDH levels is crucial in better understanding the behavior of CSC during therapy. Here, we analyzed the basal levels of ALDH^+^ cells through flow cytometry of four MEC cell lines. We found that cell lines have a different percentage of CSC varying from 1.08 to 3.25% (Fig. 4A). UM-HMC-1 and UM-HMC-3B presented a significantly higher percentage of ALDH positive cells, above 3%, compared to all other cell lines (p < 0.01).

Next, we aim at evaluating the effects of Emetine and Vorinostat as single agents over the population of ALDH^+^ cells. We observed that both drugs were capable of reducing the population of ALDH^+^ cells from all tested cell lines (Fig. 4B). Interestingly, Vorinostat produced a major reduction of both UM-HMC-1 and UM-HMC-3B compared to Emetine (Fig. 4C). Vorinostat was also more efficient than Emetine in the overall depletion of CSC within all cell lines (Fig. 4D).

### Combined inhibition of NFκB and global acetylation of histones is an efficient strategy to eradicate MEC cancer cells and its CSC

Treatment with a single agent provides a competitive advantage to tumor cells to engage resistance-associated pathways and thrive during therapy. The strategy of combined therapy can minimize drug resistance by eliminating cells that are singly resistant to either drug thus enhancing treatment efficacy. Following our positive results using Vorinostat and Emetine as single agents, we decided to explore the potential benefit of administering both agents to MEC. Our strategy takes into consideration the influence of the chromatin on cellular response to external factor^45^ and its ability to repair DNA^46^. We have shown that a compacted chromatin architecture (hypoacetylated) plays a major role in drug resistance^18^. For this reason, we decided to sensitize MEC cells using the HDAC inhibitor Vorinostat followed by the inhibition of the NFκB pathway using Emetine.

**Figure 5:**
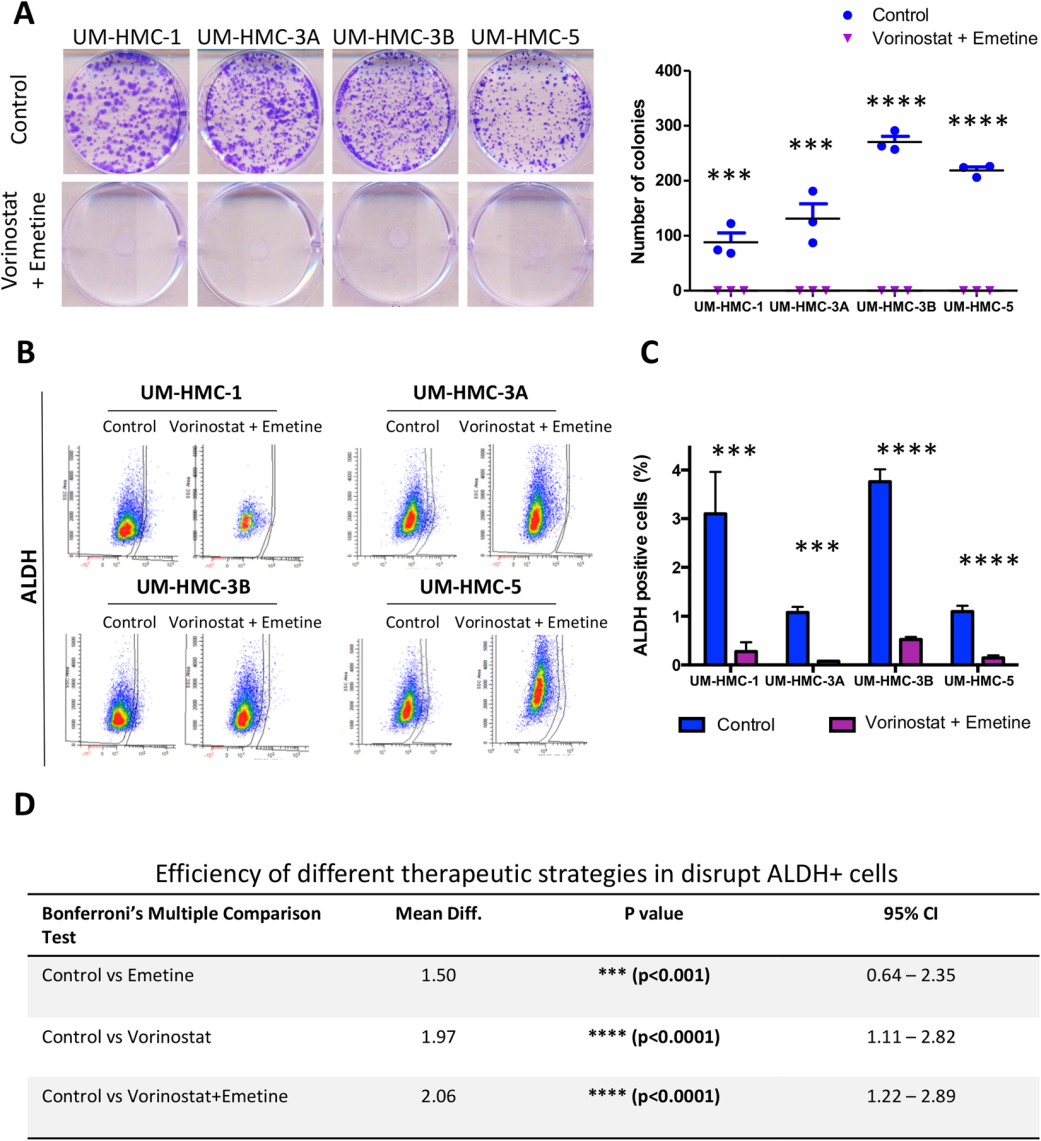
Combined therapy provide supperior results in reducing tumor cells and CSC. (**A**) Representative images of colonies fixed and stained with 0.1% crystal purple. Note that combined treatment led to a complete disruption of colony formation only in all cell lines. Colony quantification (colonies having >50 cells were counted as surviving colonies) revealed that after combined therapy the number of colonies was reduced to zero in all cell lines. (**B**) Combined therapy significantly reduced the population of ALDH^+^ cells in all MEC cell lines (p < 0.01). (**C**) Comparing the reduction of ALDH^+^ positive cells after the different treatment modalities, we observed that Control vs. Vorinostat+ Emetine demonstrated a higher mean difference, revealing that this treatment achieved the most relevant results. (**D**) The efficiency of each therapeutic strategy in depleting CSC. Note that administration of Vorinostat shows similar results to combined administration of Vorinostat and Emetine (****p < 0.0001) (mean difference 2.06 - Vorinostat/Emetine and 1.97 - Vorinostat).

Initially, we analyzed the effect of combined therapy on the MEC cells clonogenic ability. Interestingly, we found that combined therapy resulted in a complete impairment of tumor cells in generating colonies in all analyzed cell lines (Fig. 5A) (***p < 0.001). Following, we evaluated the effects of combined therapy over the population of CSC. We observed a significant reduction of CSC population in all analyzed MEC cell lines (Fig. 5B and C). Interestingly, when comparing the efficiency of combined therapy to disrupt the population of CSC (ALDH+ cells) with the administration of single agents (Vorinostat or Emetine), we found no significant difference between combined therapy and the administration of Vorinostat alone (Fig. 5D). Both conditions were able to efficiently disrupt the population of ALDH+ cells when compared to ALDH levels in the control group (****p < 0.0001) (mean difference 2.06 - Vorinostat/Emetine and 1.97 - Vorinostat). Although the administration of Emetine alone did significantly reduce the population of ALDH+ cells, it was less effective than combined therapy or administration of Vorinostat alone (***p < 0.001) (Fig. 5D).

**Figure 6:**
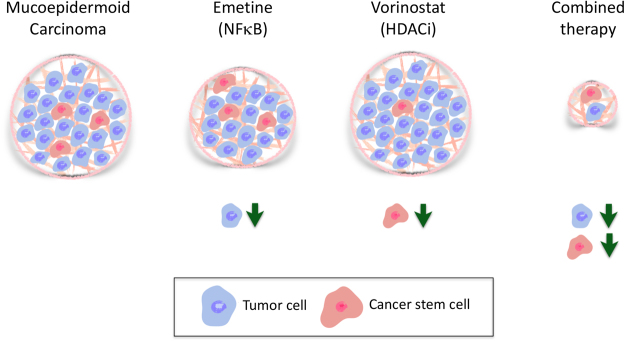
Schematic representation of the main findings of the study. MEC comprises a population of more differentiated cells, which account for the majority of cells in the bulk of the tumor, and a small population of highly tumorigenic and more undifferentiated cells, known as CSC. Emetine acts on tumor cells and can significantly reduce the viability of the majority of MEC cells. Nevertheless, the population of CSC demonstrates enhanced resistance to Emetine compared to non-CSC tumor cells. Vorinostat present high efficiency in depleting MEC CSC, but lower efficiency in disrupting non-CSC tumor cells. Combined administration of Vorinostat and Emetine provide superior results on the population of CSC and non-CSC compared to single agent therapy.

In summary, Emetine showed better results in reducing the surviving fraction of tumor cells, while Vorinostat was efficient in depleting CSC. Combined, both drugs were able to efficiently disrupt the population of CSC while reducing overall viability of mucoepidermoid carcinomas (Fig. 6).

## Discussion

Advanced cases of MEC are associated with poor prognosis, especially due to the lack of effective systemic therapies capable of eradicating tumor cells. In fact, most of the anticancer drugs act on rapidly dividing cells. Therefore the indolent growth of most SGC represents an obstacle for available therapy^8^. In the past two decades, the paradigm for cancer treatment evolved from conventional and nonspecific drugs to highly selective, mechanism-based therapies^47^. This new and promising approach is based on targeted inhibition of key pathways involved in tumor development and progression. Outstanding progress has been achieved using targeted therapies like in the case of leukemias^48^, breast cancer^49^, and non-small-cell lung cancer^50^. Unfortunately, this is not true for SGC, where we observe that cisplatin or cisplatin-based regimens remain the most common systemic therapies employed^8^. A significant limitation of single-agent targeted therapies is the development of tumor resistance^19–21^. Treating advanced tumors with a single agent often result in the selective enrichment of resistant tumor cells driven in part by the acquisition of new mutations^19^. These complications can be overcome by targeting distinct pathways. In the present study, we demonstrated that by targeting histone deacetylases and the NFκB signaling pathway we efficiently disrupted tumor cells and its population of CSC.

Salivary gland tumors contain a heterogeneous cellular population comprised of ductal, acinar-like cells and intermediate cells that are often associated with increased aggressive behavior. The identification of molecular pathways involved in tumor progression and resistance to therapy is essential to tailor an effective therapy. Towards this goal, we have identified the NFκB as a highly active signaling pathway in MEC^17^. Moreover, we demonstrated that NFκB inhibition downregulates p21 expression in MEC cell lines. Although p21 activation is traditionally associated with cell cycle arrest, recent studies demonstrated that p21 expression is associated with cell proliferation, transformation, and poor prognosis^51–54^. Along with p21 downregulation, we observed that NFκB inhibition was capable to induced MEC cells apoptosis^17^. These results indicate that NFκB signaling pathway is required for MEC cell proliferation and tumor progression. Therefore, we propose that NFκB pathway represents a promising target capable of disrupting MEC progression. In fact, a single dose of Emetine had a significant impact on the surviving fraction of MEC cells. The success of novel therapeutic approaches also depends on the ability to disrupt all subpopulations of tumor cells including the slow cycling cells. Such strategy includes targeting of the subpopulation of CSC, which present high tumorigenic potential.

The theory of CSC postulates that only a limited number of cells within the tumor mass are endowed with self-renewal properties, and present resilience to therapy being capable of colonizing distant sites of the body. Such virtues are extremely relevant as it correlates to treatment failure followed by tumor recurrence and metastasis. Although exciting, it is important to keep in perspective some limitations with the available technique to identify CSC^55^. One of the major problems is the poor characterization of the population of stem cells in the tissues that develop solid tumors^55^. Such reduced information directly impacts the identification and availability of stem cell markers to CSC researcher of solid tumors. Yet, several surface markers have been proposed in the literature including CD24 for pancreatic and lung cancer, CD44 for breast, liver and head and neck cancers, CD133 (or Prominin) used for brain, colorectal, lung and liver cancers, and EpCAM/ESA for colorectal, and pancreatic cancers^55^. To overcome potential biases using surface markers to identify CSC, we choose to use the enzymatic activity of ALDH. ALDH is found highly expressed in stem cells from different cell types^56–59^, and highly expressed in the population of tumor stem cells^40–42,56,60^. Therefore, ALDH activity may be used as a common marker for both normal and malignant stem and progenitor cells.

It’s important to keep in mind that the methods used herein can identify cells presenting high tumorigenic potential. It appears that CSC might present some fluidity and several theories have been postulated to explain CSC origin. In the hierarchical CSC model, CSC originate from undifferentiated cells and progress to terminally differentiated cancer cells during tumorigenesis, in a similar manner to that observed in embryology^61,62^. Evidence now available suggests a stochastic or fluid process governing the acquisition of a “stemness” potential by cancer cells. According to this concept, CSC might originate from both progenitor cells or differentiated cells that have the potential to re-activate self-renewal machinery once stimulated by specific factors. This theory postulate that the vast majority of differentiated cells can be reprogrammed to a more undifferentiated stage, however, only a few cells can be efficiently reprogrammed into a stem cells^61,63^. Another theory, known as the elite model, postulates that just a limited portion of cells, usually more primitive, are competent for reprogramming^63^. More studies are necessary to clarify CSC development process; nevertheless, its therapeutic relevance is well established^12–17^.

In MEC we observed that CSC represent around 1–3% of all neoplastic cells. An important characteristic of CSC is its chromatin status. The chromatin configuration is modulated by how tightly DNA is spooled around histones and the dynamic changes that occur in this structure are mainly driven by histone acetylation and histone deacetylation^45^. DNMT-1 triggers histone deacetylase activity^64^ and suppress cell differentiation^65^, leading to a stem-cell phenotype. Moreover, compacted chromatin is associated with poor DNA accessibility to drugs. We have previously demonstrated that chemoresistant head and neck cancer cells present more compacted chromatin^18^. Based on these facts, we believe that sensitizing MEC with an HDAC inhibitor, such as Vorinostat, can induce CSC differentiation and enhance chromatin accessibility. We observed that Vorinostat as a single agent is efficient in depleting CSC (ALDH^+^ cells) in all MEC cell lines. Additionally, when Vorinostat is associated with a subsequent dose of Emetine, depletion of CSC is enhanced. NFκB inhibition is likely to influence the population of CSC through epithelial-mesenchymal transition (EMT) of cancer cells as recently described by Dong and colleagues^66^. They have shown that knockdown of NFκB in HeLa cells resulted in the inhibition of Bmi1, Sox2, and Oct4 and increased E-cadherin expression^66^. In fact, we have recently shown that histone acetylation decreases during oral carcinogenesis accompanied by an increase in EMT markers^67^, suggesting that in more advanced cases tumor cells are characterized by a more compact chromatin with greater invasive capacity. Therefore, the therapy proposed herein for MEC patients might present effects beyond CSC reduction as the inhibition of the EMT process.

Overall, we provide a novel and promising therapeutic strategy to manage MEC using two FDA-approved drugs targeting the NFκB pathway and by inducing global chromatin acetylation.

## Methods

### Cell lines

MEC cell lines^10^ UM-HMC-1 (p. 89, minor salivary gland), UM-HMC-3A (p.124, minor salivary gland-local recurrence), UM-HMC-3B (p. 126, minor salivary gland-lymph node metastasis), and UM-HMC-5 (p. 129, minor salivary gland-radiation resistant cells/pair of UM-HMC3A)^17^. Cell lines were maintained in a 5% CO_2_ humidified incubator at 37 °C and cultured in RPMI 1640 (Thermo Scientific, Waltham, MA, USA) supplement with 10% of Fetal Bovine Serum (Thermo Scientific), 1% antibiotic (Invitrogen, Carlsbad, CA, USA), 1% L-glutamine (Invitrogen) 20 ng/ml epidermal growth factor (Sigma–Aldrich), 400 ng/ml hydrocortisone (Sigma–Aldrich), 5 µg/ml insulin (Sigma–Aldrich). Cells were treated with Vorinostat (Cayman Chemical Company Ann Arbor, MI, USA) and Emetine dihydrochloride hydrate (Sigma–Aldrich). IC50 of Vorinostat and Emetine were previously established for each cell lineage using MTT assay in monolayer adhered cells^16,17^. DNA genotyping by short tandem repeat (STR) profiling was performed independently by Biosynthesis Inc. (Lewisville, TX, USA). All MEC cells achieved 100% of identity-based on 15 autosomal short tandem repeat loci and in gender identity locus (amelogenin).

### Tumorsphere formation assay

MEC cells lines were plated on ultra-low attachment 6 well plate. Sphere formation was observed daily. To evaluate the ability of sphere formation under histone H3 acetylation conditions and NFκB inhibition, Vorinostat or Emetine were administered on the first day of culture for the monotherapy groups or on the first and second day respectively in the combined group. Spheres growing in suspension were collected at day 5 and transferred to a glass slide by centrifugation at 1500 rpm, 4 °C for 10 minutes using a cytospin system, following by eosin & hematoxylin staining or fixation with paraformaldehyde 4% in PBS for 15 min at RT to further be processed for immunofluorescence.

### Immunofluorescence

Blockage was performed with 0.5% (v/v) Triton X-100 in PBS and 3% (w/v) bovine serum albumin (BSA). Cells were then incubated with anti-p65/NFkB (BD Bioscience, Mountain View, CA, USA). Cells were then washed three times and incubated with TRITC-conjugated secondary antibody and stained with Hoechst 33342 for visualization of DNA content. Images were taken using a QImaging ExiAqua monochrome digital camera attached to a Nikon Eclipse 80i Microscope (Nikon, Melville, NY, USA) and visualized with QCapturePro software.

### Clonogenic survival assay

For the clonogenic assay, cells were plated into 6-well cell culture plates in a concentration previously determined by the plating efficiency. After overnight incubation, cells were treated with Vorinostat or Emetine. Cells were allowed to grow for additional 7 days to form colonies before stained with 0.1% crystal purple. Colonies that presented >50 cells were counted as surviving colonies.

### Flow Cytometry

MEC CSC cells were identified by flow cytometry for ALDH (aldehyde dehydrogenase) activity. The Aldefluor kit (StemCell Technologies, Durham, NC, USA) was used according to the manufacturer’s instructions to identify cells with high ALDH enzymatic activity. Cells with or without pretreatment as indicated in individual experiments were suspended with activated Aldefluor substrate (BODIPY-amino acetate) or negative control (dimethylamino benzaldehyde, a specific ALDH inhibitor) for 45 minutes at 37 °C. All samples were analyzed in a FACS Canto IV (BD Biosciences) at the University of Michigan Flow Cytometry Core.

### Statistical analysis

All statistical analysis was performed using GraphPad Prism (GraphPad Software, San Diego, CA). Statistical analysis of the mitosis assay, Ki67 staining and flow cytometry were performed by one-way analysis of variance (ANOVA) followed by Tukey’s multiple comparison tests. Asterisks denote statistical significance (*p < 0.05; **p < 0.01; ***p < 0.001; ****p < 0.0001; and NS *p* > 0.05).

### Data Availability

The datasets generated during and analyzed during the current study are available from the corresponding author on reasonable request.
